# The prevalence and risk factors associated with Leptospira in donkeys in Ngaka Modiri Molema District, North West Province, South Africa

**DOI:** 10.14202/vetworld.2020.2020-2027

**Published:** 2020-09-28

**Authors:** Kibambe Kiayima Daddy, Mulunda Mwanza, James Wabwire Oguttu, Lubanza Ngoma

**Affiliations:** 1 Department of Animal Health, School of Agriculture, Faculty of Natural and Agriculture Sciences, North West University, Private Bag X2046, Mmabatho 2735, South Africa; 2 Department of Agriculture and Animal Health, College of Agriculture and Environmental Sciences, University of South Africa, Florida Science Campus, Johannesburg, South Africa

**Keywords:** donkeys, leptospirosis, risk factors, seroprevalence

## Abstract

**Background and Aim:**

Leptospirosis is one of the major emerging global economic and health problems affecting donkeys, thereby reducing their work output. Furthermore, the disease has public health importance because of its zoonotic nature. Despite the significant contribution donkeys make to the national economy, less attention is given to diseases that afflict donkeys and reduce their productivity and performance. A cross-sectional study was conducted to investigate the seroprevalence of Leptospira and identify risk factors associated with the occurrence of the disease among donkeys in the study area.

**Materials and Methods:**

A questionnaire survey was used to collect the following data: Demographic, environmental, management, and health-related factors. Blood samples were aseptically collected from 365 randomly selected donkeys from 19 villages. The sera were tested using the microscopic agglutination test. Categorical variables were summarized and presented as proportions and their 95% confidence interval (CI). A binary logistic regression model was fitted to the data to identify risk factors associated with Leptospira seroprevalence in donkeys within the study areas.

**Results:**

The majority of the donkeys (29.6%; n=108/365) were from Mafikeng local municipality, and the rest (19.7%; n=72/365) were from Ratlou. Just over half (58.1%; n=212/365) of the donkeys tested were female, and the remaining (41.9%; n=153/365) were males. In addition, most donkeys (42.7%; n=156/365) were between 6 and 12 years old, followed by those between 0 and 5 years (37%; n=135/365), and only 20.3% (n=74/365) were above 12 years. Out of the donkeys tested, 11.5% (95% CI: 4.86-18.14) donkeys tested positive for Leptospira antibodies. The most common serovar was Bratislava (81%; n=34/42), followed by Tarassovi (19.04%; n=8/42). While gender was positively associated with seroprevalence of the disease (Adjusted Odds Ratio [AOR]=4.88; p=0.0001), the presence of horses (AOR=0.226; p=0.002) and agricultural activities (AOR=0.093; p=0.0001) in the vicinity of the dwellings of the donkeys were negatively associated with Leptospira seropositivity in the study area.

**Conclusion:**

Findings reported here show that donkeys in the study area are reservoirs for the predominant serovar Bratislava and the less dominant serovar Tarassovi. The gender of the donkey was a risk factor for Leptospira seroprevalence. Further studies are needed to investigate the role of agricultural activities in the vicinity of the dwellings of donkeys on the occurrence of Leptospira in the study area.

## Introduction

More than 97% of donkeys are found in developing countries and are kept for working purposes [1]. In South Africa, donkeys are among the most important domestic animals because of their socio-economic importance among resource-limited communities in rural areas, where modern means of transportation are limited, unaffordable, and/or inaccessible [2].

Leptospirosis is one of the major emerging global economic and health problems affecting donkeys; thereby reducing their work output [3-5]. The disease is caused by any of the 250 identified pathogenic serovars grouped within the former pathogenic species *Leptospira interrogans* sensu lato [6,7]. Donkeys become infected when they come into contact with water or soil that has been contaminated by urine from carrier animals. Transmission is mainly influenced by exposure to several risk factors, such as inadequate management practices and poor environmental conditions [8]. Clinical signs in infected animals include fever, renal and hepatic injuries, pulmonary hemorrhage, reproductive failure, and periodic ophthalmia [9,10]. Studies on leptospirosis in cattle [11], pigs [12], dogs [13], and horses [14] across South Africa suggest that agglutinating antibodies may occur in healthy animals that are infected with host-adapted serovars. However, it is likely that these animals represent only a fraction of likely hosts.

The infecting serovars and risk factors associated with the seropositivity in donkeys have not yet been extensively investigated in South Africa. Despite their significant contribution to the national economy, less attention is given to diseases that afflict donkeys and reduce their productivity and performance. Therefore, the aim of this study was to estimate the seroprevalence of *Leptospira* serovars in the study area using the microscopic agglutination test (MAT). The study also investigated risk factors associated with seropositivity in donkeys.

## Materials and Methods

### Ethical approval and informed consent

Ethical clearance number (NWU-00182-18-S5) for this study was issued by the Animal Care, Health and Safety in Research Ethics Committee (AnimCare) of the North-West University. In addition, the Act 35 (Animal Diseases Act) of 1984, Section 20 approval to perform research on animals and collect samples, was obtained from the Department of Agriculture, Forestry and Fisheries (DAFF): Directorate of Animal Health (Reference Number 12/11/1/3) of the Republic of South Africa. Only participants who filled in the consent form participated in the study. Furthermore, participants were informed that they were free to withdraw from the study without any consequences.

### Study area

**Figure-1:**
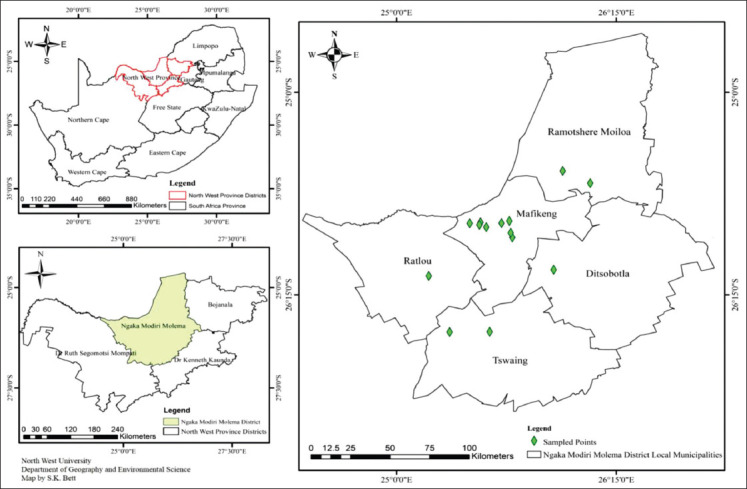
Map showing the sampling sites in the five local municipalities of NMMD.

The study was carried out in the Ngaka Modiri Molema District (NMMD) of the North West Province (Figure-1), which borders Botswana. According to DAFF, as of 2016, NMMD was one of the areas in South Africa with a large donkey population (Unpublished data). The district is divided into five local municipalities. The vegetation type comprises two major biomes: The grassland and the savanna biome [15]. According to the South African Weather Services [16], rainfall in the study area is erratic and varies from an average of 600 to 700 mm/annum. Temperature varies from very hot in summer (daily average 32°C) to mildly cold in winter (monthly average minimum of up to 12°C).

### Study design

A cross-sectional study design was adopted to realize the objectives of this study. A two-stage sampling procedure was adopted across NMMD of the North West Province. A total of 19 villages were randomly selected, from which 365 donkeys were selected using systematic random sampling for bleeding to collect blood samples. Sampling was carried out between March 2017 and October 2018.

### Sampling size and sampling strategy

According to DAFF (unpublished data), there were approximately 6,125 donkeys in the NMMD in 2016. Based on this population size, the sample size of 365 donkeys was estimated using EpiInfo version 6.04 software (CDC, Atlanta, GA, USA) at a 95% confidence and 5% margin of error.

### Data collection

Structured interviews using a questionnaire were used to collect data on possible risk factors. The questionnaire included both closed and open-ended questions on risks factors for infection with leptospirosis such as demographic, geographic, and environmental factors, as well as management practices. Only respondents who consented to participate voluntarily and who owned donkeys were invited to participate in the study. Whole blood samples were aseptically collected in a vacutainer tube. Sera were separated and stored at 4°C before shipping on ice to the Agricultural Research Council-Onderstepoort Veterinary Institute (ARC-OVI) laboratory for serological testing using MAT.

### Serological testing using the MAT

The MAT was performed at ARC-OVI laboratory using the microplates method with a panel of eight reference *Leptospira* serovars: Bratislava, Tarassovi, Canicola, Hardjo type, Icterohaemorrhagiae, Szwajizak, Pomona and Grippotyphosa,. The eight reference *Leptospira* serovars were representative of the eight main serogroups known to exist in Southern Africa [14,17], including those known to be maintained by donkeys elsewhere [18]. All the tested serovars were maintained in EMJH liquid medium and a cutoff titer of ≥1:100 was used as the conclusive criterion for seropositivity [10].

### Statistical analysis

All data were captured into a single Microsoft Excel file and exported to the Statistics Package for the Social Sciences software (SPSS) version 20.0 (IBM Corp., Armonk, NY, USA). Before analysis, data were assessed for inconsistencies and missing values. Relative frequencies (proportions) with their 95% confidence interval (CI) were calculated for categorical variables.

The univariate logistic regression model was fitted to identify variables for inclusion in the multivariable model at a generous p≤0.20 (Annexure-A). There afer, a multivariate binary logistic regression model was fitted using manual backward selection, using all variables that had univariate associations (p<0.20). Confounding was assessed in the final model by observing changes in the effect measures when a variable was removed from the model. If there was a change of more than 10% in effect measures of the other variables, the variable in question was considered a confounder and was thus retained in the model. The odds ratio (OR) and their corresponding 95% CI were computed. The Hosmer-Lemeshow goodness-of-fit test was used to assess the model fit for the final model. Statistical significance was assessed at α=0.05.

## Results

### Summary statistics

Table-1 presents the summary of the demographic, geographic, and management factors. Out of the 365 donkeys recruited in this study, the majority (29.6%) were from Mahikeng Local Municipality, while 80.3% were from the medium rainfall zone.

**Table-1:** Distribution of donkeys by demographic, environmental, and management factors (n=365).

Variables	Level	^#^Number (%)	*95% CI
Demographic factors			
Gender			
	Male	153 (41.9)	31.2-51.9
	Female	212 (58.1)	45.9-70.3
Age			
	0-5 years	135 (37)	25.1-48.9
	6-12 years	156 (42.7)	30-55.5
	≥12	74 (20.3)	11.5-29.1
Breed			
	Locale breed	364 (99.7)	80.1-119.2
	Cross breed	1 (0.3)	0.77-1.3
Ecological zone			
	Average annual rain fall		
	Low	72 (19.7)	11.0-28.3
	Medium	293 (80.3)	62.7-97.8
Agricultural activities			
	Maize	100 (30.7)	17.1-37.6
	Fruit and vegetables	39 (12)	4.2-17.1
	None	187 (57.4)	42.5-42.5
Municipalities of origin			
	Mafikeng	108 (29.6)	18.9-40.2
	Ratlou	72 (19.7)	10.7-28.3
	Tswaing	83 (22.7)	13.4-32
	Ditsobotla	30 (8.2)	2.5-13.8
	Moilwa	72 (19.7)	0.7-28.3
	Management factors		
Water source			
	River	115 (31.5)	20.5-42.5
	Municipality water	60 (16.4)	8.1-24.3
	Dam	39 (10.7)	4.2-17.1
	Borehole	54 (14.8)	7.2-22.3
	Borehole and dam	97 (26.6)	16.5-36.1
Donkeys’ use			
	Racing	14 (3.8)	0.0-7.6)
	Working	339 (92.9)	74-111.8
	Other	12 (3.3)	0.2-6.8
Other animal in vicinity			
Cattles			
	Yes	66 (18.1)	9.7-26.4
	No	299 (81.9)	64.1-99.6
Horses			
	Yes	74 (20.3)	11.4-29.1
	No	291 (79.7)	61.6-96.5
Pigs			
	Yes	10 (2.7)	0.5-5.9
	No	313 (96.3)	77.6-116.6
Sheep and goat			
	Yes	55 (15.1)	10.4-22.7
	No	310 (84.9)	66.8-102.9
Presence of rodent on farm			
	Yes	253 (71.3)	54.7-87.8
	No	102 (28.8)	18.26-39.31

* 95% CI=95% Confidence interval,

# Column totals might not add up to 365 due to missing data

Just over half (58.1%) of the donkeys were females, while the rest were males. The majority of the donkeys (42.7%) were between 6 and 12 years old, followed by 0-5 year old (37%) (Table-1). Almost all the donkeys (92.9%) included in this study were kept for working purposes. In terms of the environment, 27.4% of the donkeys were sampled from areas that were in close proximity to maize planted in the vicinity of homestead, as the main agricultural activity. The rest (10.7%) were from areas that were within the vicinity of fruit and vegetable gardens. In addition, just over 70% (71.3%) of owners reported the presence of rodents on their farms (Table-1).

### *Leptospira* seroprevalence in the NMMD

Out of the 365 donkeys tested, 11.5% (42/365) tested positive for *Leptospira* antibodies. Most positive reactors recorded titer values of 1:100 or 1:200 (Table-2).

**Table-2:** Distribution of *Leptospira* serovars based on the MAT titers.

Serovars	Titers						*Tot [AP] ^#^AP 95% CI		

	1:100	1:200	1:400	1:800	1:1600	1:3200			
Bratislava	18	12	3	1	-	-	34 [81]	63.36	98.64
Tarassovi	2	3	-	-	-	3	8 [19.04]	10.48	27.52
Canicola	-	-	-	-	-	-	0 [00]	0.00	0.00
Hardjo type	-	-	-	-	-	-	0 [0.00]	0.00	0.00
Icterohaemorrhagiae	-	-	-	-	-	-	0 [0.00]	0.00	0.00
Szwajizak	-	-	-	-	-	-	0 [0.00]	0.00	0.00
Pomona	-	-	-	-	-	-	0 [0.00]	0.00	0.00
Grippotyphosa	-	-	-	-	-	-	0 [0.00]	0.00	0.00
Overall	20	15	3	1	0	3	42		

* Tot [AP]=Total and Proportion of Agglutination positive isolates,

# AP 95% CI=95% Confidence Interval for proportions of Agglutination positive isolates. MAT=Microscopic agglutination test

Only two serovars (representative of two serogroups) were identified, and of these, the most common *Leptospira* serovar was Bratislava 81% (95% CI: 63.3-98.6), followed by Tarassovi at 19.04% (95% CI: 10.4-27.5) (Table-2).

### Inferential statistics

#### Risk factors for donkey exposure

The assessment of simple associations using the univariate analysis identified municipality (p<0.0001), gender (p=0.0093), average annual rainfall (p<0.001), agricultural activities (p<0.0001), the sources of water (p<0.0001), presence of rodents (p<0.0001), presence of horses (p=0.1), and sheep and goats in the vicinity (p=0.063) based on a liberal p=0.20 as being significantly associated with seropositivity (Annexure A). Therefore, these variables were included in the multivariable model.

In the final multivariable logistic regression model (Table-3), the odds of a male donkey testing positive for *Leptospira* antibodies were 5 times (Adjusted OR (AOR)=4.88; 95% CI: 2.01-11.82; p≤0.0001) that of female donkeys. If a donkey resided in the vicinity of fruit and vegetable gardens, the odds of such a donkey testing positive were lower (AOR=0.09395%; CI: 0.031-0.27; p≤0.0001;) than for donkeys that were reared in homesteads that did not have a garden. Similarly, if a donkey was kept near a maize garden, it had lower odds (AOR 0.74; 95% CI: 0.27-2; p=0.573) of being seropositive, compared to donkeys that were found on homesteads without a garden in the vicinity. In addition, if a donkey was co-grazed with horses, the odds of testing seropositive (AOR=0.226; 95% CI: 0.089-0.57 p≤0.002) were low, compared to donkeys that were not co-grazed or did not live in the vicinity of horses.

**Table-3:** Results of the multivariate logistic regression analysis: Assessment of significant risk factors for *Leptospira* seropositivity.

Variables	Tested (n)	Positive n (%)	OR (95% C I)	p-value
Gender				
Female	212	19 (10)	Referent	
Male	153	23 (15)	4.8 (2.01-11.8)	<0.0001
Agricultural activities				
Maize	100	15 (15)	0.74 (0.27-2)	0.573
Fruits and vegetables	39	10 (25.6)	0.09 (0.03-0.2)	<0.0001
None	187	9 (4.8)	Referent	
Horses in vicinity				
Yes	74	13 (17.5)	0.22 (0.08-0.5)	0.002
No	262	29 (11)	Referent	

## Discussion

The present study presents findings of circulating serovars of *Leptospira* among donkeys in NMMD, North West Province, South Africa. Two serovars, Bratislava serovar and Tarassovi serovar, were identified. The male donkeys were more likely to test positive for *Leptospira* antibodies when compared to the female donkeys. We also observed that donkeys found within the vicinity of fruit and vegetable gardens or maize gardens were less likely to test seropositive for the disease, compared to those reared in homesteads without a garden. Likewise, donkeys that were co-grazed with horses, had a lower risk of testing seropositive, compared to donkeys that were not co-grazed, or did not live in the vicinity of horses.

In a study conducted in Mexico by Alvarado-Esquivel *et al*. [18], the authors reported a markedly high seroprevalence of 77.8% (n=151/194) among healthy donkeys. Similarly, Benkirane *et al*. [19] observed a seroprevalence of 60% (n=15/9) in donkeys in Morocco. This sharply contrasts with 11.5% (n=42/365) seroprevalence observed in this study. However, our findings are closer to those reported by Grubišic *et al*. [20], who observed a 25.53% seroprevalence in Croatia. Climatic factors, along with the MAT cutoff used in the different studies, may be responsible for the observed disparity [21] between our study and the Mexico study by Alvarado-Esquivel *et al*. [18], who reported a higher prevalence. Adler and de la Peña Moctezuma [10] showed that variations in the seroprevalence can be between 1% and 95% in horses, depending on the geographical location and serovars assessed.

Similar to the Moroccan study by Benkirane *et al*. [19], we observed antibodies against the Australis serogroup (Table-1). However, while in NMMD the Australis serogroup, *L. interrogans* serovar Bratislava was the predominant serovar, in the Moroccan study Benkirane *et al*. [19] observed that *L. interrogans* serovar Australis was more predominant. The difference between the two studies notwithstanding, since the *L. interrogans* serovar Australis was not included in the MAT panel used in this study, the authors are not able to conclude whether there was a difference between the predominant serovar within the Australis serogroup observed in the present study and the Morocco study conducted by Benkirane *et al*. [19]. However, consistent with the findings reported in this study, Grubišic *et al*. [20] also observed that *L. interrogans* serovar Bratislava was the most prevalent serovar in Croatia.

Other studies have reported contrasting findings to those of the present study. For example, in Iran, Grippotyphosa and Icterohaemorrhagiae were the most prevalent serovars [22]. In Egypt Butembo, Pomona, Icterohaemorrhagiae, and Canicola were the most prevalent [23], while in Mexico, Icterohaemorrhagiae, and Sejroe were the most prevalent [18]. This disparity may be attributed not only to the difference in serovars included in the MAT panel, but also due to the cutoff used to interpret the results of different studies.

In South Africa, the serovar Bratislava has also been reported to cause subclinical disease in pigs [12,24], horses [14], cattle [11], and dogs [13]. These findings show that serovar Bratislava appears to be widespread countrywide and is probably maintained by donkeys in the NMMD, North West Province of South Africa.

It was observed that donkeys reared within the vicinity of fruit and vegetable farming as the main agricultural activity, were significantly less likely to test seropositive for *Leptospira* (AOR=0.093; 95% CI: [0.031-0.27; p≤0.0001), compared to those that were not reared in the vicinity of an agricultural activity (Table-3). Similarly, donkeys that were within the vicinity of a maize garden were less likely to test seropositive (AOR 0.74; 95% CI 0.27-2; p=0.573), compared to donkeys that were not in close vicinity of a maize garden. This is contrary to what was expected because agricultural activities, such as fruit and vegetable fields that are irrigated with water, tend to be contaminated by rodent urine and hence act as reservoirs for *Leptospira* infections for donkeys living in close proximity to such fields. This is supported by the findings by Azócar-Aedo *et al*. [25], who reported that animals like cats that dwell in places where agricultural activities were carried out, are 30 times more likely to be seropositive to *Leptospira*. This was also consistent with findings in the study by Alavi and Khoshkho [26], who observed similar findings among rice farmers in Iran.

In the present study, it was observed that male animals were 5 times (AOR=4.88; 95% CI: 2.01-11.82; p≤0.0001) more likely to test positive than female donkeys (Table-3). This is in conformity with findings reported by Alvarado-Esquivel *et al*. [18] in donkeys, and those of Kikuti *et al*. [27] in dogs. Kikuti *et al*. [27] also reported gender as a risk factor for *Leptospira* in dogs (OR=0.439, 95% CI: 0.62-0.89; p=0.009). The authors of the present work are of the view that the most plausible explanation for the results reported here, is that male donkeys are usually more engaged in working activities, and therefore spend a lot of time outside of their premises where they are exposed to the infected environment (areas). The latter increases the risk of exposure to *Leptospira* infection among male donkeys.

However, there are studies that have reported findings that are contrary to those observed in the present study. For example, some authors observed that there was no statistically significant differences between the proportions of *Leptospira* infection in males and females [28,29].

Several studies have identified horses as reservoirs of serovar Bratislava around the world [30], and in South Africa [14]. In view of this, the fact that donkeys in this study that were living in the vicinity of horses and co-grazing with horses were at a lower risk of testing seropositive (AOR=0.226; p≤0.002; 95% CI: [0.089-0.57], was not expected. This is important considering that the serovar Bratislava was predominant among donkeys living in the vicinity of horses, and that according to Schoonman and Swai [31], co-grazing different species increases the exposure risk resulting in the spread of the disease among species.

## Conclusion

This study concluded that within the serogroup Australis, Bratislava was the predominant *Leptospira* serovars in donkeys in the study area, and that donkeys may act as reservoirs for *Leptospira* bacteria. This makes donkeys in the study area a potential source of infection for humans and other animals. Furthermore, the study found that only gender was a risk factor for donkeys testing seropositive for Leptospira, while having horse in the vicinity, and living in the vicinity of fruit and vegetable gardens, and other farming activities, were protective factors for *Leptospira* seroprevalence. However, given the limited nature of this study, these findings should be interpreted with caution. In light of this, there is a need for larger studies to further understanding of the risk factors for *Leptospira* infection in donkeys in the study area.

## Author’s Contributions

KKD collected samples, conducted the experiments, analyzed the data, and drafted the manuscript. LN conceived and supervised the project and edited the manuscript. MM co-supervised the project and helped in drafting of the manuscript. JWO extensively reviewed the manuscript and analyzed the data. All authors read and approved the final manuscript.

## Annexure

**Annexure A:** Assessment of simple associations using the univariate analysis (p-value of 0.20).

Variables	No. of tested	Number positive (%)	χ^2^	p-value
Demographic factors			3.216	0.0093
Gender				
Male	153	23(15)		
Female	212	19(9)		
Age			3.4112	0.193
0-5 years	135	18(13)		
6-12 years	156	20(15)		
12 years and above	74	4(5)		
Breed				
Locale breed	364	42(42)		
Cross breed	1	0(0)		
Donkey’s use				
Other	12	0(0)		
Working	339	37(11)		
Racing	14	5(35.7)		
Geographic &environmental factors				
Temperature				
Low	0	0(0)		
Medium	365	42(11)		
High	0	0(0)		
Avg annual rainfall			11.66	0.001
Low	0	0(0)		
Medium	293	42(14.3)		
High	0	0(0)		
Agricultural activities			40.957	<0.0001
Fruits & veg	39	10(26)		
Sugarcanne	0	0(0)		
Maize farma	100	15(15)		
None	187	9(5)		
Missing	39	-		
Municipality of origin				
Mafikeng	108	26(24)		
Ratlou	72	0(0.00)		
Tswaing	83	15(18.07)		
Ditsobotla	30	1(3.44)		
Moilwa	72	0(0.00)		
Management factors				
Water sources			38.67	<0.0001
River	115	0(0.00)		
Municipality	60	4(6)		
Dam	39	13(33.3)		
Borehole	54	14(18.5)		
Borehole & dam	97	15(15.4)		
Others animals in vicinity				
Cattle			0.359	0.67
Yes	66	9(13.63)		
No	299	33(11)		
Horse			3.348	0.1
Yes	74	13(17)		
No	291	29(10)		
Pig				
Yes	10	0(0.00)		
No	355	42(13.4)		
Sheep and goat			3.932	0.063
Yes	55	2(3.63)		
No	310	40(13)		
Dog			0.011	1
Yes	133	15(11.27)		
No	232	17(11.63)		
Rodent			10.843	<0.0001
Yes	253	39(15.41)		
No	102	3(3)		
Disease clinical signs			1.114	0.291
Ocular problem				
Yes	22	1(4.54)		
No	343	41(12)		
Abortion			-	-
Yes	0	0(0.00)		
No	118	10(8.47)		
Stillbirth			-	-
Yes	0	0(0.0)		
No	118	10(8.47)		
Vaccination history			-	-
Yes	0	(0.00)		
No	210	14(6.66)		
Antibiotic treatment			-	-
Yes	0	0(0.00)		
No	330	34(10.3)		

* Column subtotals may not sum to the total due to the missing data
